# QLiS-SF: Development of a short form of the quality of life in schizophrenia questionnaire

**DOI:** 10.1186/s12888-017-1307-1

**Published:** 2017-04-27

**Authors:** T. Senin, M. Franz, M. Deuschle, N. Bergemann, J. Kammerer-Ciernioch, M. Lautenschlager, T. Meyer

**Affiliations:** 1 Integrative Rehabilitation Research Unit, Institute on Epidemiology, Social Medicine and Health Systems Research, Hannover Medical School, Carl-Neuberg-Str. 1, 30625 Hannover, Germany; 2 Vitos Klinikum Giessen-Marburg, Licherstraße 106, 35394 Giessen, Germany; 3 Centre for Psychiatry, Justus Liebig University, Giessen, Germany; 4 Central Institute of Mental Health Mannheim, Faculty of Medicine Mannheim, University of Heidelberg, Square J 5, 68159 Mannheim, Germany; 5 Saxon Hospital Rodewisch, Center for Psychiatry, Psychotherapy, Psychosomatics, and Neurology, Bahnhofstraße 1, 08228 Rodewisch, Germany; 6 Klinik für Allgemeine Psychiatrie und Psychotherapie Ost, Klinikum am Weissenhof, 74189 Weinsberg, Germany; 7 Department of Psychiatry and Psychotherapy Charité Berlin, Charitéplatz 1, 10117 Berlin, Germany

**Keywords:** Schizophrenia, Quality of life, Short form

## Abstract

**Background:**

There is a need for useful standardized Quality of Life (QoL) measures for people diagnosed with schizophrenia. Therefore, a short form of the self-administered Quality of Life in Schizophrenia (QLiS) scale was developed and validated.

**Methods:**

Four steps were taken to develop the abridged version using samples from the Clinical Analysis of the Treatment of Schizophrenia (CATS) study. Firstly, a model with second order scales was developed using exploratory factor analysis (EFA). Secondly, it was tested in an independent sample using confirmatory factor analysis (CFA). Thirdly, this model served as the basis for selecting items for the short form. Distributional properties, content reviews, and factor loadings were taken into account in this step. Fourthly, the resulting short form was validated through confirmatory factor analysis (CFA). Composite reliability scores were calculated for the new subscales.

**Results:**

Three second order scales were constructed: illness-related quality of life (QoL), social life and finances, and global subjective well-being. CFA of the new theoretical model resulted in a CFI of 0.67 and absolute fit indices of CMIN/df = 2.55, RMSEA = 0.08, SRMR = 0.09. The selected 13 items showed good statistical properties and good fit of content to subscale. Fit of the underlying theoretical model with the reduced number of items was tested in an independent sample. Absolute and fit indices of the short form model were satisfactory (CFI = 0.95, CMIN/df = 2.23, RMSEA = 0.06, SRMR = 0.04). Composite reliability scores for three subscales were above 0.70.

**Conclusions:**

The short form of the QLIS (QLiS-SF) showed good model fit and reliability. It should only be considered for use if the application of the long version is not suitable.

**Electronic supplementary material:**

The online version of this article (doi:10.1186/s12888-017-1307-1) contains supplementary material, which is available to authorized users.

## Background

The term schizophrenia, as defined by current nosologies, represents a heterogeneous syndrome, with multiple likely etiologies, pathological forms and courses. The diagnosis of schizophrenia comprises a broad spectrum of partly severe states, often characterized by a wide spectrum of psychopathological symptoms (positive and negative symptoms, general psychopathology), as well as illness trajectories ranging between recovery and severe states of chronicity. In addition, people often have to deal with substantial side effects of psychotropic treatments. Due to early age of onset, tendency for recurrence, chronic course, as well as for persistent impairment of cognitive and social functions, people diagnosed with schizophrenia may experience significant impairments of their life perspectives. Schizophrenic disorders contribute to a strong burden on health systems [1]. Thus, persons diagnosed with schizophrenia might be confronted with a high level of impact of the disorder and its consequences on their quality of life (QoL). The evaluation of medical and psychosocial interventions should therefore include measures of QoL [2, 3]. In addition, QoL improvement has proven to be an important predictor for symptomatic remission and functional recovery in patients diagnosed with schizophrenia [4]. Moreover, from a clinical perspective, QoL is considered an essential part of patient management [5]. Further uses for QoL assessments are evaluative and outcome research [6], as well as identifying patients’ preferences and facilitating communication in clinical practice [7]. Interpretation of the results of QoL measurements in persons diagnosed with schizophrenia is difficult due to the perceived impact of depressive and psychotic symptoms, poor insight, and cognitive deficits. Clinician or assessor rated measures of QoL show only moderate correlation with self-ratings [8]. The WHO defines QoL as the individuals’ perception of their position in life in the context of the culture and values systems in which they live and in relation to their goals, expectations, concerns, and standards [9]. The evaluation of medical and psychosocial interventions should therefore include measures of subjective QoL that have a high content-validity [2, 3, 6, 10]. In QoL research a distinction is made between so-called disease-specific measures of QoL which tend to be more responsive and clinically useful, and generic QoL measures which do not focus on any specific condition and have the advantage of enabling comparisons of QOL across diseases [11]. In this regard, disease-specific QoL measures based on patients’ perceptions of their own lives should be used [10] to gain adequate information on the various problems actually experienced by people with a diagnosis of schizophrenia [12], acknowledging that the term schizophrenia does not relate to a circumscribed clinical disease but rather represents a spectrum of different conditions and etiologies. Although some QoL scales were developed, tested, and applied in patients with a diagnosis of schizophrenia (see [4, 10]) there is still a need for useful standardized QoL measures for patients with this diagnosis [13].

The Quality of Life in Schizophrenia (QLiS) scale is a self-administered QoL instrument for people who have been diagnosed with a schizophrenic disorder according to ICD 10. It was developed in a thorough stepwise process [10] in people treated in psychiatric hospitals, psychiatric practices or sheltered hostels and workshops. In total, more than 700 persons diagnosed with schizophrenia were included in the developmental process [6, 10]. A wide spectrum of people from different care settings with different levels of disability were included (as described in more detail elsewhere [6, 10]), from day clinics, acute and long-term hospital wards, community based settings such as sheltered or independent housing, with a broad range from normal to sheltered work to no work and from daily structuring activities to vocational therapy, vocational rehabilitation up to normal vocational status. A high level of content validity was achieved by integrating patient’s views and preferences into the whole development process. Accordingly, the QLiS is based on structured open ended interviews. These were conducted with patients diagnosed with schizophrenia (*N* = 268) to discover their understanding of QoL and what is essential for their QoL. The interview consisted of open questions about what had made them happy during the last week, what made them feel happy in general, what aspects of life they would find very hard to renounce, and what their understanding of QoL was (see [6, 10]). These questions did not selectively address any specific treatment modalities or settings. They were conducted either in a clinical setting or at the person’s living environment. A content analysis was performed on patients’ responses and a comprehensive model of QoL was developed. A 130-item pilot version was developed based on studies on quantification of theme and item importance as well as an analysis of item specificity. Further developmental stages consisted of empirical analyses of response scale formats, item selection, construction of subscales, and testing of reliability and validity [10]. Besides a full description of the developmental process [10], results on discriminant and convergent validity were also reported [6].

The final QLiS questionnaire comprises 52 items in 12 subscales (Table 1) and two additional items related to one’s work situation depending on whether they have some sort of work or respective rehabilitation programme, or are unemployed or without any respective work activity. The item response format has four response categories (“disagree”, “rather disagree”, “rather agree” and “agree”). The test-retest-reliabilities and internal consistencies of 11 of the 12 subscales were above 0.70 [6, 10]. Analysis of discriminant validity showed that QLiS subscales are sufficiently distinct from other QoL instruments [6].

**Table 1:** Subscales of the QLiS^a^

Subscale		Subscale	
	Items		Items
Social contacts	5	Abilities to manage daily life	4
Appreciation by others	4	Appraisal of accommodation/housing	5
Relationship to family	3	Financial situation	4
Appraisal of pharmacotherapy	6	Leading a ‘normal’ life	3
Appraisal of psychopathological symptoms	6	Confidence	4
Cognitive functioning	5	General life satisfaction	3
		*Work or rehabilitation situation* ^a^	2 (1 + 1)

^a^Including 2 work-related items, either of them is used and its result reported, therefore these two items do not constitute a regular subscale

QoL measures remain underutilized in clinical practice despite their indicated need and utility [14]. This might be related to time constraints [15]. In this context the development of short-forms is very important. An abridged version of the QLiS could support the feasibility of the QLiS, especially in surveys, clinical studies in which QoL is not a primary outcome, or even in the clinical setting in the follow-up of the treatment course. Therefore, the aim of this study was to develop a short form of the QLiS based on psychometric and substantive grounds.

## Methods

### Data

Data were drawn from two samples of the Clinical Analysis of the Treatment of Schizophrenia (CATS) study [16]. CATS was a non-interventional pharmacoepidemiological study following a naturalistic design. The study was developed to characterize key areas of treatment such as psychopathology, medication, adverse drug reactions, cognitive function, QoL, sexual functions, and parameters of the metabolic syndrome of a large and representative group of patients who had been diagnosed with schizophrenia [16] (Table 2). *N* = 512 people completed the QLiS. It was conducted in 49 German in- and outpatient hospital departments. Its aim was to evaluate the treatment of patients diagnosed with schizophrenia as found in current clinical practice. Study inclusion criteria were schizophrenia spectrum disorder (all ICD-10 F2-diagnoses), age 18 years or older, and given informed consent [17]. All procedures performed were in accordance with the ethical standards of the 1964 Helsinki declaration and its later amendments. Informed consent was obtained from all individual participants included in the study. The CATS study comprised the assessment of QoL at two time-points, namely at the start of treatment (t1) and after 4 weeks (t2) by means of the QLiS [9]. For the present study we used both the sample at t1 and t2. We divided the cross-sectional sample (t1) randomly in two independent and approximately equal sized samples. Therefore, three samples, two from t1 and one from t2, were used for the analyses presented.

**Table 2:** Socio-demographic and clinical characteristics of the three samples

	Sample 1	Sample 2		Sample 3
	Cross-sectional sample – first half	Cross-sectional sample – second half	Significance of difference between sample 1 & 2 (*χ* ^*2*^ test or *t*-test)	Longitudinal sample
Sample size	*n* = 251	*n* = 261		*n* = 364
Sex (% male)	61%	55%	0.185	59%
Age (in years)	38.3 (± 11.5)	39.9 (± 11.6)	0.132	39.0 (± 11.7)
Family status			0.862	
Single	73%	69%		72%
Married	14%	18%		15%
Divorced/separated	11%	12%		12%
Widowed	1%	1%		1%
Education			0.329	
None	5%	8%		5%
Special education	1%	2%		1%
Intermediate	53%	58%		57%
Technical high school	2%	3%		3%
University entrance qualification	33%	23%		27%
Unknown	6%	7%		7%
Vocational status			0.612	
No work	38%	34%		37%
Sheltered work	11%	11%		12%
Normal work status	22%	20%		20%
Retired worker	21%	30%		25%
Other	9%	5%		7%
Age at onset of illness	28.2 (± 9.2)	29.0 (± 10.1)	0.346	28.3 (± 9.5)
Duration of illness (in years)	9.5 (± 9.2)	10.6 (± 9.6)	0.229	10.1 (± 9.5)
Global Assessment of Functioning (GAF)	48.6 (± 15.1) range 15–90	48.3 (± 16.1) range 6–91	0.855	55.6 (± 14.4) range 22–90

### Statistical methods

**Fig. 1:**
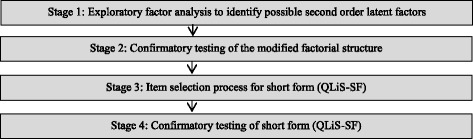
Stages of the development of the QLiS short form (QLiS-SF)

Since the QLiS was designed as a profile-instrument, there is no global score that represents the QoL of persons with schizophrenia by a single number. When shortening the QLiS the underlying conceptual model had to be modified. In the QLiS, all subscales were represented by a small number of items ranging from 3 to 6 items. We decided not to represent each subscale by only one or two items because reliability and representation of the respective subscale domains were expected to become insufficient. Therefore, we decided to relate the items of the QLiS to second order factors. This allows a sufficient distinction of QoL aspects as well as an adequate number of items for a sufficient level of reliability. This idea of a second order factor structure has been applied in the widespread SF-36 instrument and the respective SF-12 short version structure [18]. A process comprising four steps or stages was set up to develop the short form (Fig. 1). The principles described in the literature were followed [19, 20].

In the first stage, an exploratory factor analysis (EFA; principal axis with varimax rotation) was performed on the scores of the twelve QLiS subscales to examine the underlying structure with respect to second order latent traits. Kaiser-Meyer-Olkin (KMO) measure and Bartlett’s test of sphericity were calculated to assess the suitability of the correlation matrices for factor analysis. A KMO value above 0.60 and a significant sphericity test indicate suitability [21].

In a second phase, the identified factorial structure was tested through confirmatory factor analysis (CFA) based on the single items by means of structural equation modelling. The following indices were calculated as recommended by Kline [22]: The absolute fit indices chi-square (*χ* ^*2*^) test, the root mean square error of approximation (RMSEA), the standardized root mean square residual (SRMR), and, as an incremental fit index, the comparative fit index (CFI). Analyses were performed using maximum-likelihood estimation. Missing values were imputed using the expectation maximization algorithm.

The *χ* ^*2*^ test compares the observed and the estimated covariance matrices. Since the test is determined by sample size and number of observed variables, the normed *χ* ^*2*^ will be reported. A normed *χ* ^*2*^ smaller 2.0 is considered as good, a number between 2.0 and 5.0 as acceptable fit of the covariance matrices. The RMSEA represents how well the model fits the population. Lower values, such as below 0.08, represent better fit of the model. An advantage of the RMSEA is the possibility of confidence interval construction. The SRMR describes the standardized difference between observed and predicted correlations. A SRMR over 0.10 suggests a fitting problem. The CFI compares the sample covariance matrix to a baseline model (independence model). Values above 0.90 are associated with good fit [23].

The third stage comprised the item selection process. It aimed to preserve both good psychometric properties and aspects of content validity of the resulting second order factors. For each item, distributional properties (items with lower means and more symmetric distributions are regarded to be more informative), factor loadings (representing the fit of the items to their respective scale), and correlations between items (to prevent the selection of highly correlated, i.e. information redundant items) supported the selection process as decision-criteria. From a perspective of content validity, we analyzed to which degree each item was able to represent the new second order subscale. How were the different items taken together able to represent the different facets of the new second order factor (i.e. especially the first order subscales of the original version of the QLiS)? Priority was given to items that seemed to be specific for the QoL-experience of persons with schizophrenia. Item selection was done using item-by-item-interpretation of all these aspects and properties taken together.

In stage four, the new second order factorial structure was tested in the t2 sample of the CATS project. Composite reliability scores for the newly developed factors were estimated for the evaluation of scale reliabilities.

All analyses were done using the Statistical Package for Social Sciences (SPSS, Version 22) and AMOS Graphics (Version 22).

## Results

### Sample characteristics

Table 2 shows the socio-demographic and clinical characteristics of the samples. We included 251 patients for the analyses in stage 1 (EFA), 261 patients in stage 2 and 3 (i.e. for CFA and item selection), and 364 patients in stage 4 for the confirmatory testing of the short form. The characteristics of the samples (Table 2) were similar in all demographic variables as well as in age at onset, duration of illness, and global assessment of functioning (GAF). Most of the participants were single, had a comparable level of education, and an average age of 38.3 to 39.9 years. There was a higher proportion of male participants in all three samples. The proportion of missing values regarding all QLiS items was very low (ranging from none to 0.8% per single item) in all three samples.

### Stage 1: Results of EFA

An exploratory factor analysis with principal axis factoring was conducted with the mean scores of the 12 subscales. KMO measure of sampling adequacy was 0.89, while Bartlett’s test of sphericity was significant (*χ* ^*2*^ = 1448, *df* = 66, *p* < 0.001). The number of latent traits was determined by using the scree-test, which indicated a three factor solution (Table 3). The three factors altogether explained 55.8% of the variance. Items were assigned to the three resulting factors by examining varimax rotated factor loadings. All items had factor loadings greater 0.50 on their associated factor. One subscale (confidence) showed cross-loading (*r* > 0.50) on two factors. It was allocated to the factor with higher loading which was also associated with a more appropriate fit from a content perspective of the new scale. The subscales were labeled according to the common characteristics of the items loading on the respective factor. The following second order scales resulted from this process: illness-related QoL, social life and finances, and global subjective well-being. Table 3 shows the associated subscales and loadings for each new scale.

**Table 3:** Loadings of the QLiS-subscales on the second order factors

Scales	Loadings		
	factor 1	factor 2	factor 3
Factor 1 Illness-related QoL			
Appraisal of pharmacotherapy	0.548		
Appraisal of psychopathological symptoms	0.710		
Cognitive functioning	0.821		
Abilities to manage daily life	0.777		
Factor 2 Social life and finances			
Appreciation by others		0.559	
Social contacts		0.553	
Relationship to family		0.558	
Appraisal of accommodation/housing		0.598	
Financial situation		0.534	
Factor 3 Global subjective well-being			
Leading a ‘normal’ life			0.702
Confidence	*(0.513)*		0.585
General life satisfaction			0.688

### Stage 2: Results of CFA

**Fig. 2:**
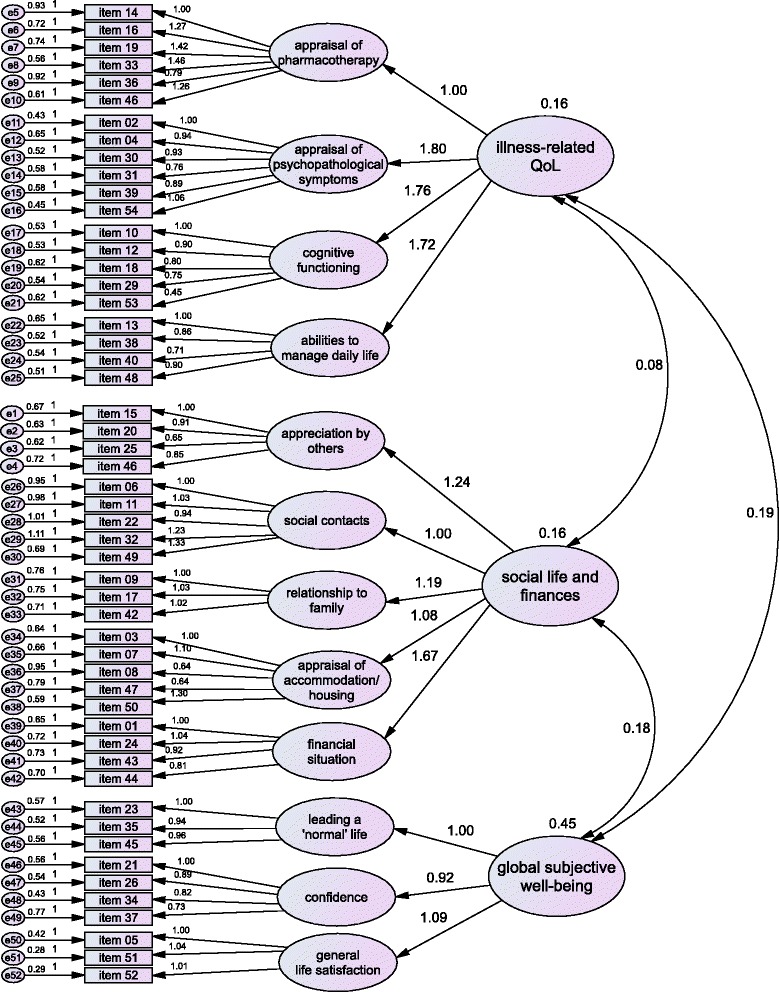
Full model with second order scales

A CFA was run with all three levels of the measurement model (items, subscales, and second order scales) to confirm the factor structure that emerged in the EFA. The resulting intercorrelation of the three second order scales was low (*r* = 0.08 - *r* = 0.19). Path coefficients between second order scales and related subscales were high and significant (Fig. 2). Absolute and incremental fit indices of the model are shown in Table 4.

**Table 4:** Results of the CFA on the original long version (stage 2) and on the final short version (stage 4)

*χ* ^*2*^ (*df*/*t*)	*p*	CMIN/df	CFI	RMSEA	SRMR
Stage 2: 3-factor-model of the original QLiS (52 items)					
3240.205	<0.001	2.549	0.670	0.077 (0.074–0.080)	0.0894
Stage 4: 3-factor-model of the new short version of the QLiS (13 items)					
138.341	<0.001	2.231	0.952	0.058 (0.045–0.071)	0.0430

### Stage 3: Results of the item selection process

All items were characterized by distributional properties (with mean score and standard deviation), loadings from items to subscales, correlations between items within subscales, and content reviews (see methods section). Thus, the selection criteria took statistical properties and reflections on content validity into account. Based on these characteristics, we discussed each item separately as a possible candidate for the short form.

As a result 22 items of the QLiS were selected. Our original goal was to end up with a smaller number of items ranging from 12 to 15. Using CFAs, based on modification indices, and issues of content validity, items were removed step by step until good measurement models with a total of 13 items and good model fit remained (see supplementary table [Additional file 1]).

### Stage 4: Results of confirmatory testing of short form

**Fig. 3:**
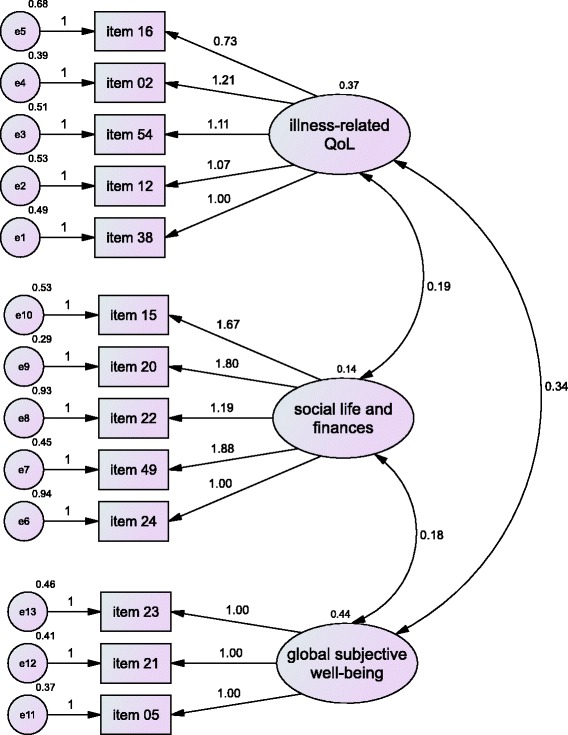
Confirmatory testing of short model

The shortened QLiS version with 13 items and its underlying conceptual model is shown in Fig. 3. Table 5 lists items of second order scales.

**Table 5:** Translation^a^ of related items of second order scales

Illness-related QoL	Social life and finances	Global subjective well-being
My medications are making me slow I often feel depressed and glum I suffer from distressing thoughts I have trouble concentrating I feel dependent on others	I feel impaired by other people (e.g. recklessness, dishonesty, untrustworthiness) I feel rejected by many people I perceive my sex life as unfulfilled I feel lonely and alone I have too little money for basic things (e.g. proper clothing, little things like cigarettes)	I lead a completely “normal life” just like other people do I look to the future with confidence I am satisfied with my life

^a^Since the original version of the QLiS is in German, these translations were made for the purpose of reporting and still have to be analyzed and validated in a transcultural adoption process. A draft version can be seen in an additional file [see Additional file 2]

The results of the CFA are shown in Table 4. Composite reliability scores for second order scales were all above 0.70 (illness-related QoL: 0.788, social life and finances: 0.729, global subjective well-being: 0.763).

## Discussion

We have presented the results of the development of a short form of the QLiS. This process ended up with a version comprising three factors that we best interpreted as illness-related QoL, social life and finances, and global subjective well-being. Psychometric properties in terms of model fit (factorial validity), item fit indices, as well as reliability (all scores >0.70) of this short form of the QLiS were favourable. The underlying theoretical model of the short form showed a sufficient fit with the data, without using correlated errors [24].

These results provide reasonable support for the quality of the QLiS short form from a psychometric perspective. However, the decision for using the short form should essentially be based on substantive issues. Here, we would like to add a note of caution. As we have argued in the introduction, there is a need for quality of life assessments in persons experiencing a chronic mental disorder, but short versions should only support the use and feasibility of QoL assessment in surveys and clinical studies in which QoL is not a primary outcome. A short form should only be considered for use if the application of the long version is not suitable. In addition, the attribution of the instrument as disease-specific might not be true in the sense that the items of the QLiS are *exclusively* important to people with a diagnosis of a schizophrenic spectrum disorder. However, the items are based solely on open questions not related to particular life domains or any therapeutic interventions. Morevoer the qualitative analyses was sensitive to the perspectives that persons diagnosed with a schizophrenic spectrum disorder had provided. By this way we have tried to be sensitive to their priorities and to relevant aspects of their lives. It has to be acknowledged, however, that the conduct of open–ended interviews within the clinical setting or the person’s living environment might have especially lead to an underrepresentation of work- or occupation related topics because they might have not been salient to the interviewees’ thoughts.

Also, the reduction of the items for the abbreviated version means a limitation imposed on the previous concept of a comprehensive profile instrument. Adhering to the conceptual model during the development of an abridged version is recommended in the literature [19]. For the short form of the QLiS, we decided not to reduce the QLiS to a very low number of items, because the main strength of the QLiS (different facets and high specificity) would have been lost. It is almost impossible to choose even fewer items without compromising the level of information. Therefore, a new conceptual model seemed more useful to obtain reliable scales, because one item per scale would not have been sufficiently informative or reliable. Last but not least, in contrast to the development of the original QLiS, patients were not directly involved in this process. However, information from patients from earlier research was used to select items for the short form [10]. Furthermore, item selection was discussed from both clinical and research-based perspectives.

For the work-domain no psychometrical sound subscale could be developed in the original QLiS, possibly due to the heterogeneity of the persons’ work situations. We know from previous studies that unemployment is still a severe problem for the majority of adults diagnosed with schizophrenia. Despite effective programs to assist with job identification and placement, research indicates that over two-thirds of adults living in the community with a diagnosis of schizophrenia are unemployed in an industrialized country [25]. A QoL-scale should be applicable to the whole group of people with a schizophrenia diagnosis, both to those who receive forms of psychosocial treatment or rehabilitation or have regular jobs and to the considerable group without work or employment. In the QLiS, the work domain might have been underrepresented to begin with, and is not represented in its short form. By the same token, the QLiS short form makes only explicit reference to one treatment modality, pharmacotherapy. Other modern therapy options such as psychotherapy, different forms of psychosocial treatment or rehabilitation, e.g. supported employment, are not explicitly mentioned but are represented in their potential impact on the life-domains included in the short form. Therefore, for the evaluation of complex psychosocial treatments, rehabilitation- or recovery-oriented interventions, users should refer to the original QLiS which provides a more comprehensive and differentiated profile.

However, we feel confident that it is better to have a psychometrically sound instrument based on a reasonable process of development of its content than to refrain from QoL assessment in surveys or clinical studies altogether. As it is true in any assessment, we have to be aware both of its strength and limitations. In case of the QLiS short form we could be confident to capture general appraisals in three global life domains important to people diagnosed with schizophrenia. We would encourage its use both in survey and outcomes research that is primarily related to the *group* of people diagnosed with schizophrenia (as opposed to the individual level) and in situations in which the application of the long version is not suitable.

## Conclusions

The QLiS-SF can give a general overview of the individual perspective on QoL of persons diagnosed with schizophrenia. The original QLiS should be used for precise measurement and more accurate classification of subjective QoL in persons with a diagnosis of schizophrenia. However, a short form reduces efforts associated with administration of QoL assessments. Therefore, short forms are likely to increase the number of assessments of important QoL domains [26]. The QLiS-SF could be a relevant alternative to the long form especially for research purposes. It has limitations with regard to the width of the interrogated areas regarding the array of evidence-based modalities of modern psychiatric rehabilitation in favor of the reliability of the short subscales. Acceptability of the long QLiS version can be assumed to be very good. This is demonstrated by extremely low proportions of missing data. Short-forms of scales are frequently associated with better acceptability [27], which could further increase acceptance of the QLiS items in the short form. Therefore, the QLiS-SF contributes to the assessment of subjective QoL in persons with a diagnosis of schizophrenia in psychiatric research. It must demonstrate its usefulness in future applications.

## Additional files

Additional file 1: Process of item selection based on modification indices and considerations of content validity (supplementary table describing the process of stepwise item elimination). (DOCX 19 kb)

Additional file 2: QLiS-SF Quality of Life in Schizophrenia Questionnaire - Short Version (draft translation of the German short version, which has to be analyzed and validated in a transcultural adoption process). (DOC 59 kb)

Additional file 3: Ethics committees that approved the multicenter study (list of all ethics committees with addresses that approved the multicenter study, from which data were drawn). (DOCX 19 kb)

## Abbreviations

CATS: Clinical Analysis of the Treatment of Schizophrenia
CFA: Confirmatory Factor Analysis
EFA: Exploratory Factor Analysis
QLiS: Quality of Life in Schizophrenia
QLiS-SF: Qualify of Life in Schizophrenia – Short Form
QoL: Quality of Life
