# Supplementary material for: QLiS-SF: Development of a short form of the quality of life in schizophrenia questionnaire
Source: BMC Psychiatry. 2017 Apr 27;17:149. doi: 10.1186/s12888-017-1307-1 (PMC5408441; doi:10.1186/s12888-017-1307-1)
Supplement: Supplementary file 1 — Process of item selection based on modification indices and considerations of content validity (supplementary table describing the process of stepwise item elimination). (DOCX 19 kb) [file 12888_2017_1307_MOESM1_ESM.docx]

Process of item selection based on modification indices and considerations of content validity

Supplementary Table Process of elimination from 22 to 13 items

| *χ²* (*df*/*t*) | *p* | CMIN/df | CFI | RMSEA | SRMR |
| --- | --- | --- | --- | --- | --- |
| 22 items | | | | | |
| 516,074 | p<.001 | 2.505 | 0.823 | 0.076 (0.068-0.084) | 0.0752 |
| 21 items: after eliminating item 18 | | | | | |
| 449,733 | p<.001 | 2.418 | 0.839 | 0.074 (0.065-0.083) | 0.0726 |
| 20 items: after eliminating item 25 | | | |  |  |
| 393,840 | p<.001 | 2.358 | 0.855 | 0.072 (0.063-0.082) | 0.0712 |
| 19 items: after eliminating item 32 | | | |  |  |
| 329,217 | p<.001 | 2.210 | 0.877 | 0.068 (0.058-0.078) | 0.0664 |
| 18 items: after eliminating item 7 | | |  |  |  |
| 296,820 | p<.001 | 2.249 | 0.882 | 0.069 (0.059-0.080) | 0.0658 |
| 17 items: after eliminating item 17 | | | |  |  |
| 248,109 | p<.001 | 2.139 | 0.901 | 0.066 (0.055-0.078) | 0.0613 |
| 16 items: after eliminating item 40 | | | |  |  |
| 192,752 | p<.001 | 1.908 | 0.925 | 0.059 (0.046-0.072) | 0.0566 |
| 15 items: after eliminating item 53 | | | |  |  |
| 171,586 | p<.001 | 1.972 | 0.929 | 0.061 (0.048-0.075) | 0.0562 |
| 14 items: after eliminating item 50 | | | |  |  |
| 119,599 | p=.001 | 1.616 | 0.958 | 0.049 (0.032-0.064) | 0.0484 |
| 13 items: after eliminating item 30 | | | |  |  |
| 97,790 | p=.003 | 1.577 | 0.963 | 0.047 (0.028-0.064) | 0.0467 |
