# Supplementary material for: QLiS-SF: Development of a short form of the quality of life in schizophrenia questionnaire
Source: BMC Psychiatry. 2017 Apr 27;17:149. doi: 10.1186/s12888-017-1307-1 (PMC5408441; doi:10.1186/s12888-017-1307-1)
Supplement: Supplementary file 2 — QLiS-SF Quality of Life in Schizophrenia Questionnaire - Short Version (draft translation of the German short version, which has to be analyzed and validated in a transcultural adoption process). (DOC 59 kb) [file 12888_2017_1307_MOESM2_ESM.doc]

# QLiS-SF

# Quality of Life in Schizophrenia Questionnaire - Short Version


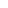


Please always refer to your **current accommodation** (clinic, dorm, own apartment etc.) and on your **life situation of the last 7 days**!***☜***

| 1. I often feel depressed and glum | strongly disagree  ⭘ | somewhat disagree  ⭘ | somewhat agree  ⭘ | strongly disagree  ⭘ |
| --- | --- | --- | --- | --- |
| 1. I am satisfied with my life | strongly disagree  ⭘ | somewhat disagree  ⭘ | somewhat agree  ⭘ | strongly disagree  ⭘ |
| 1. I have trouble concentrating | strongly disagree  ⭘ | somewhat disagree  ⭘ | somewhat agree  ⭘ | strongly disagree  ⭘ |
| 1. I feel impaired by other people (e.g. recklessness, dishonesty, untrustworthiness) | strongly disagree  ⭘ | somewhat disagree  ⭘ | somewhat agree  ⭘ | strongly disagree  ⭘ |
| 1. My medications are making me slow | strongly disagree  ⭘ | somewhat disagree  ⭘ | somewhat agree  ⭘ | strongly disagree  ⭘ |
| 1. I feel rejected by many people | strongly disagree  ⭘ | somewhat disagree  ⭘ | somewhat agree  ⭘ | strongly disagree  ⭘ |
| 1. I look to the future with confidence | strongly disagree  ⭘ | somewhat disagree  ⭘ | somewhat agree  ⭘ | strongly disagree  ⭘ |
| 1. I perceive my sex life as unfulfilled | strongly disagree  ⭘ | somewhat disagree  ⭘ | somewhat agree  ⭘ | strongly disagree  ⭘ |
| 1. I lead a completely “normal” life just like other people do | strongly disagree  ⭘ | somewhat disagree  ⭘ | somewhat agree  ⭘ | strongly disagree  ⭘ |
| 1. I have too little money for basic things (e.g. proper clothing, little things like cigarettes) | strongly disagree  ⭘ | somewhat disagree  ⭘ | somewhat agree  ⭘ | strongly disagree  ⭘ |
| 1. I feel dependent on others | strongly disagree  ⭘ | somewhat disagree  ⭘ | somewhat agree  ⭘ | strongly disagree  ⭘ |
| 1. I feel lonely and alone | strongly disagree  ⭘ | somewhat disagree  ⭘ | somewhat agree  ⭘ | strongly disagree  ⭘ |
| 1. I suffer from distressing thoughts | strongly disagree  ⭘ | somewhat disagree  ⭘ | somewhat agree  ⭘ | strongly disagree  ⭘ |
