# Supplementary material for: QLiS-SF: Development of a short form of the quality of life in schizophrenia questionnaire
Source: BMC Psychiatry. 2017 Apr 27;17:149. doi: 10.1186/s12888-017-1307-1 (PMC5408441; doi:10.1186/s12888-017-1307-1)
Supplement: Supplementary file 3 — Ethics committees that approved the multicenter study (list of all ethics committees with addresses that approved the multicenter study, from which data were drawn). (DOCX 19 kb) [file 12888_2017_1307_MOESM3_ESM.docx]

**Ethics committees that approved the multicenter study**

Ethics committee of the Medical Faculty, University Mannheim

Theodor-Kutzer-Ufer 1-3
68167 Mannheim

Germany

Ethics committee of the Medical Faculty, Ruprecht-Karls-University Heidelberg

Alte Glockengießerei 11/1

69115 Heidelberg

Germany

Ethics committee of the Medical Faculty, Charité Berlin

Charité - Universitätsmedizin Berlin

Charitéplatz 1

10117 Berlin

Germany

Ethics committee of the Medical Faculty, Heinrich Heine University Düsseldorf

Moorenstr. 5

40225 Düsseldorf

Germany

Ethics committee of the Medical Faculty, Friedrich-Alexander-University Erlangen-Nürnberg

Krankenhausstraße 12

91054 Erlangen

Germany

Ethics committee of the Medical Faculty, [Albert](https://webmail.mh-hannover.de/owa/redir.aspx?SURL=wHwPpBRX3BVJ93TNrxT6OMPqQJ6QOTWQUWXXLMiK6GA0UaIb7W3UCGgAdAB0AHAAOgAvAC8AdwB3AHcALgBkAGkAYwB0AC4AYwBjAC8AZQBuAGcAbABpAHMAYwBoAC0AZABlAHUAdABzAGMAaAAvAEEAbABiAGUAcgB0AC4AaAB0AG0AbAA.&URL=http%3a%2f%2fwww.dict.cc%2fenglisch-deutsch%2fAlbert.html) [Ludwig](https://webmail.mh-hannover.de/owa/redir.aspx?SURL=Nwv9_CD2hl1vjvyEggq4VPbI7UVc6pglweaaZ5nNMpg0UaIb7W3UCGgAdAB0AHAAOgAvAC8AdwB3AHcALgBkAGkAYwB0AC4AYwBjAC8AZQBuAGcAbABpAHMAYwBoAC0AZABlAHUAdABzAGMAaAAvAEwAdQBkAHcAaQBnAC4AaAB0AG0AbAA.&URL=http%3a%2f%2fwww.dict.cc%2fenglisch-deutsch%2fLudwig.html) [University](https://webmail.mh-hannover.de/owa/redir.aspx?SURL=SH_abZziF15LggXRJ6_L3aQQtjYqnfE9p6Jv6yN_pHE0UaIb7W3UCGgAdAB0AHAAOgAvAC8AdwB3AHcALgBkAGkAYwB0AC4AYwBjAC8AZQBuAGcAbABpAHMAYwBoAC0AZABlAHUAdABzAGMAaAAvAFUAbgBpAHYAZQByAHMAaQB0AHkALgBoAHQAbQBsAA..&URL=http%3a%2f%2fwww.dict.cc%2fenglisch-deutsch%2fUniversity.html) [of](https://webmail.mh-hannover.de/owa/redir.aspx?SURL=oPQy0DhO0JX4985QO8k1ZWwad6xWQKCDFBvblqtLU-c0UaIb7W3UCGgAdAB0AHAAOgAvAC8AdwB3AHcALgBkAGkAYwB0AC4AYwBjAC8AZQBuAGcAbABpAHMAYwBoAC0AZABlAHUAdABzAGMAaAAvAG8AZgAuAGgAdABtAGwA&URL=http%3a%2f%2fwww.dict.cc%2fenglisch-deutsch%2fof.html) Freiburg

Engelberger Straße 21

79106 Freiburg

Germany

Ethics committee of the Medical Faculty, Justus Liebig University Giessen

Alte Frauenklinik

Klinikstr. 32

35392 Gießen

Germany

Ethics committee of the Medical Faculty, Friedrich-Schiller-University Jena

Bachstraße 18

07740 Jena

Germany

Ethics committee of the Medical Faculty, University Leipzig

Käthe-Kollwitz-Straße 82

04109 Leipzig

Germany

Ethics committee of the Medical Faculty, Ludwig-Maximilian-University Munich

Pettenkoferstr. 8a

80336 Munich

Germany

Ethics committee of the Medical Faculty, Westphalian Wilhelm University Münster

and of the State Chamber of Physicians of Westphalia

Gartenstr. 210 – 214

48147 Münster

Germany

Ethics committee of the Landesärztekammer (Medical Chamber) of Baden-Wuerttemberg

Jahnstraße 40

70597 Stuttgart

Germany

Ethics committee of the Landesärztekammer (Medical Chamber) of Bavaria

Mühlbaurstr.16

81677 München

Germany

Ethics committee of the Landesärztekammer (Medical Chamber) of Hamburg

Weidestraße 122 b
22083 Hamburg

Germany

Ethics committee of the Landesärztekammer (Medical Chamber) of Hesse

Im Vogelsgesang 3

60488 Frankfurt am Main

Germany

Ethics committee of the Landesärztekammer (Medical Chamber) of Lower Saxony

Berliner Allee 20

30175 Hannover

Germany

Ethics committee of the Landesärztekammer (Medical Chamber) of North Rhine

Tersteegenstr. 9

40474 Düsseldorf

Germany

Ethics committee of the Landesärztekammer (Medical Chamber) of Rhineland-Palatinate

Deutschhausplatz 3

55116 Mainz

Germany

Ethics committee of the Landesärztekammer (Medical Chamber) of Saxony

Schützenhöhe 16

01099 Dresden

Germany
